# Enzymatic Saccharification Behavior and Compositional Characteristics of *Mucuna pruriens*‐Based Amazake: A Comparison With Conventional Rice Amazake

**DOI:** 10.1002/fsn3.72132

**Published:** 2026-07-17

**Authors:** You Yazaki, Takako Koriyama

**Affiliations:** ^1^ Faculty of Food and Nutritional Science Toyo University Asaka–shi Saitama Japan

**Keywords:** Amazake, *Mucuna pruriens*, rice koji, saccharification, sugar composition

## Abstract

Amazake is a traditional Japanese non–alcoholic beverage produced through enzymatic saccharification using rice koji. Conventional rice–based amazake contains high levels of glucose derived from starch saccharification, prompting interest in alternative plant–based substrates with distinct sugar compositions and nutritional characteristics. However, research on amazake produced from non–rice substrates remains limited. In this study, amazake produced entirely from *Mucuna pruriens* beans was evaluated and compared with conventional rice amazake to assess its feasibility and fundamental compositional properties. Mucuna beans were subjected to prolonged thermal treatment (90°C for 16 h), followed by homogenization, prior to saccharification with rice koji. Changes in L–3,4–dihydroxyphenylalanine (L–DOPA) content, saccharification behavior, sugar composition, and antioxidant‐related indices were examined. Preprocessing reduced the intrinsic L–DOPA content from approximately 3 g/100 g (wet basis) in raw beans to below 0.04 g/100 g, with a further decrease observed after saccharification. Although Mucuna bean amazake exhibited lower soluble solids and reduced sugar formation than rice amazake, time–course analyses of Brix, reducing sugars, and sugar composition confirmed steady saccharification. These results indicate detectable and progressive enzymatic hydrolysis despite the structural resistance of legume starch. Notably, distinct sugar distribution patterns, including low–degree–of–polymerization sugars and α–1,6–linked oligosaccharides, were observed during saccharification, highlighting qualitative differences in sugar composition beyond total sugar content. In addition, Mucuna bean amazake exhibited consistently higher antioxidant‐related indices, including DPPH radical scavenging activity, total phenolic content, and ferric reducing antioxidant power, than rice amazake throughout saccharification, likely reflecting the release of bound phenolics and generation of antioxidant peptides from the Mucuna matrix. These findings provide baseline compositional evidence supporting the feasibility of *M. pruriens* as a novel plant–based substrate for amazake production.

## Graphical abstract

*Mucuna pruriens* ‐based amazake exhibited lower sugar content, higher nutritional value, and greater antioxidant capacity than conventional rice amazake, demonstrating its potential as a novel plant‐based functional beverage.

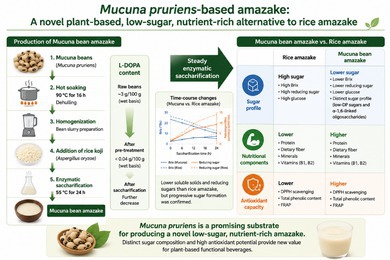

## 1 Introduction

Amazake is a traditional Japanese non–alcoholic beverage produced through the enzymatic saccharification of rice koji. In rice–based amazake, starch is hydrolyzed primarily by amylolytic enzymes derived from *Aspergillus oryzae*, resulting in a naturally sweet and nutrient–rich product containing glucose, B vitamins, free amino acids, and various minerals (Oguro et al. 2017; Yamashita 2021). In terms of composition and production process, this rice–koji amazake differs substantially from amazake made by diluting and sweetening sake lees (Kurahashi 2021). Despite its nutritional benefits, rice amazake (RA) typically contains 5–15 g of carbohydrates per 100 mL (Aso et al. 1962; Oguro et al. 2019), and may raise concerns regarding glycemic impact due to high glucose content (Santos et al. 2023; Starzyńska‐Janiszewska et al. 2024). Legume‐based substrates may exhibit distinct saccharification behavior compared with cereal substrates because their matrices contain both starch and relatively high levels of protein, which can influence enzymatic accessibility (Betancur‐Ancona et al. 2002; Zhou et al. 2004). In addition, the increasing demand for plant‐based fermented foods has stimulated interest in exploring legume‐derived substrates for traditional fermentation products (Tamang et al. 2020).

*Mucuna pruriens* (velvet bean) is an underutilized legume containing 20%–30% protein, high dietary fiber, and substantial amounts of minerals, such as iron, zinc, and magnesium, as well as essential amino acids and phenolic compounds (Siddhuraju et al. 2000). These nutritional components are generally lacking in rice, indicating that Mucuna may serve as a suitable substrate for producing nutritionally enhanced amazake. Mucuna beans naturally contain high levels of L–3,4–dihydroxyphenylalanine (L–DOPA), a catechol–type compound that causes enzymatic and non–enzymatic browning during processing (Bell and Janzen 1971; Daxenbichler et al. 1971). Therefore, effective removal or reduction of L–DOPA is essential for food applications. Previous studies have reported that soaking and boiling can markedly reduce the level of L–DOPA by leaching it into the cooking water (Teixeira et al. 2003); thus, multistep processing—as in the production of amazake—may be advantageous for reducing residual concentrations of L–DOPA.

Research on amazake produced from non–rice substrates remains limited. Moreover, because legumes differ fundamentally from rice in starch structure, cell wall composition, and phenolic content, substantial differences in saccharification kinetics and functional properties are expected.

To the best of our knowledge, no systematic compositional comparison has been reported for amazake produced entirely from Mucuna beans in terms of saccharification behavior, L–DOPA reduction, and antioxidant properties. Conventional RA therefore serves as an appropriate reference food with well‐established compositional data, whereas the compositional profile of amazake produced entirely from Mucuna beans remains largely unknown. Based on the distinct protein and carbohydrate composition of *M. pruriens* , we hypothesized that amazake produced from Mucuna beans would exhibit saccharification behavior and sugar composition different from those of conventional RA.

Given these gaps in the research, we aimed to clarify the feasibility and fundamental characteristics of amazake produced from *M. pruriens* by systematically comparing it with conventional RA. Specifically, we investigated the following aspects of Mucuna–based amazake production:

1. Reduction in L–DOPA during preprocessing and saccharification, including α‐amylase activity as a mechanistic indicator,
2. Time–course changes in soluble solids, pH, and reducing sugars as indicators of saccharification behavior, and
3. Sugar composition during saccharification, with particular attention to low–degree–of–polymerization sugars and α–1,6–linked oligosaccharides, and
4. Changes in antioxidant‐related indices, including DPPH radical scavenging activity, total phenolic content, and ferric reducing antioxidant power during saccharification.

By systematically examining these chemical and compositional changes, this study provides baseline data for evaluating the feasibility of *M. pruriens* as a plant–based substrate for amazake production and contributes foundational information for the future development of nutritionally distinctive, low–sugar amazake products.

## 2 Materials and Methods

### 2.1 Materials

Commercially cooked and packaged rice (Oishii Gohan, Koshihikari, Uonuma, Iris Ohyama Inc., Miyagi, Japan) was used as the rice substrate. The product was heated in a microwave oven (600 W, 90 s) immediately before use, according to the manufacturer's instructions. Mucuna beans ( *M. pruriens var. utilis* cv. Hassjo, 2023 harvest) were purchased from the Nasu Mucuna Farm (Tochigi, Japan). The beans were hot–soaked in reverse osmosis (RO) water at a 1:20 (w/v) ratio at 90°C for 16 h, manually dehulled, and used without additional boiling. This prolonged hot–soaking treatment was applied to facilitate L–DOPA removal and soften the bean structure prior to further processing, following the procedure described by Nagatsuka and Koriyama (2026). The prepared beans were stored at −20°C and thawed at 4°C the day before use.

In preparation for compositional analyses, raw, dried beans were ground using a milling machine (DM–6, Labonect Co., Osaka, Japan) and sieved through a 500–μm mesh.

### 2.2 Preparation of Amazake

To ensure comparable saccharification behaviors, the moisture contents of cooked rice and pretreated Mucuna beans were adjusted to be equivalent. Thawed Mucuna beans were homogenized with an equal weight of RO water using a homogenizer (Physcotron, Microtec Co., Chiba, Japan) at 7500 rpm for 15 s, followed by gentle filtration through cloth to match the initial weight. Cooked rice was hydrated with RO water to achieve the same moisture content as the Mucuna bean paste.

Amazake was prepared using a substrate: water: rice koji ratio of 2:2:1 (w/w). Rice koji was added after the thermal pretreatment of the Mucuna beans and served as the enzymatic source for saccharification. In a 100 mL glass beaker, 20 g of substrate (cooked rice or prepared Mucuna beans), 20 g of RO water, and 10 g of rice koji (Miyakouji, ISESOU Inc., Tokyo, Japan) were mixed thoroughly with a spatula. The samples were loosely covered with a plastic film and incubated in a temperature–controlled chamber (MOV–212F, SANYO Electric Co., Osaka, Japan) at 55°C for 24 h. Aliquots were collected at 0, 2, 4, 8, 16, and 24 h for analysis. Amazake samples were prepared in three independent batches following the same procedure.

### 2.3 Proximate Composition, Minerals, and Vitamins

The general composition (moisture, protein, fat, ash, carbohydrate, and dietary fiber), minerals, and vitamins of amazake samples were analyzed by an accredited external laboratory (Japan Food Research Laboratories, Tokyo, Japan) using official methods of the Association of Official Analytical Chemists. Carbohydrate content was calculated by difference.

### 2.4 L–DOPA Content

The L–DOPA content was determined using a modified extraction–high–performance liquid chromatography (HPLC) method. One gram of sample was homogenized with 35 mL of 0.4 M phosphate buffer (pH 4.0) and shaken at 20°C for 60 min. The extract was centrifuged (9000 rpm for 15 min), and the supernatant was analyzed by HPLC (EXTREMA, JASCO, Tokyo, Japan). Separation was performed on a Shodex Rspak DE–613 column (Showa Denko, Tokyo, Japan) with 0.1 M phosphate buffer (pH 2.0)/methanol (9:1) at a flow rate of 1.0 mL/min. The column temperature was maintained at 40°C. L–DOPA was detected at 200 nm (UV–4075, JASCO) and quantified using a standard (FUJIFILM Wako Pure Chemical Corp., Osaka, Japan). The results are expressed as g L–DOPA per 100 g (wet basis).

### 2.5 Measurement of Brix and pH

Amazake (2.0 g) was diluted six–fold with RO water, homogenized (7500 rpm, 15 s), and centrifuged (5000 rpm, 5 min, 4°C). The supernatant was used to determine the soluble solids content using a digital refractometer (PAL–S; Atago Co., Fukui, Japan). The pH was measured directly using a calibrated pH meter (D–51 with a 6252–10D electrode; Horiba Ltd., Kyoto, Japan).

### 2.6 Reducing Sugar Content

Reducing sugars were quantified as glucose equivalents using the Somogyi–Nelson method (Somogyi 1945; Nelson 1944). Reagents C (alkaline copper) and D (arsenomolybdate) were prepared according to the standard protocol. In brief, 0.3 mL of the sample extract and 0.3 mL of reagent C were heated in a boiling water bath for 15 min, cooled to 26°C ± 1°C room temperature, and mixed with 0.3 mL of reagent D. After adding 0.9 mL RO water, the absorbance was measured at 520 nm using a spectrophotometer (Synergy HTX, Agilent Technologies, CA, USA). Measurements were performed in triplicate and expressed on a dry–weight basis (dwb). The Somogyi–Nelson method quantifies total reducing sugars but does not differentiate between individual monosaccharides.

### 2.7 Determination of Mono–And Oligosaccharides

Mono– and oligosaccharides were quantified using HPLC (Chromaster system, Hitachi High–Tech Corporation, Tokyo, Japan). Prepared amazake samples were homogenized at 7500 rpm for 15 s and subsequently centrifuged at 10,000 rpm for 15 min at 4°C. The resulting supernatant was filtered through a 0.45 μm membrane filter. The filtrate was then diluted twofold with 50% (v/v) ethanol and filtered again through a 0.45 μm membrane filter prior to HPLC analysis. For glucose determination, samples were further diluted tenfold with 50% (v/v) ethanol to ensure that concentrations fell within the linear range of the detector.

Chromatographic separation was performed using an Asahipak NH2P–50 4E column equipped with an Asahipak NH2P–50G 4A guard column (Showa Denko K.K., Tokyo, Japan). The mobile phase consisted of acetonitrile/water (77:23, v/v) delivered at a flow rate of 1.0 mL/min using a Chromaster 5110 pump (AG–42). The injection volume was 20 μL (Chromaster 5280 autosampler), and the column temperature was maintained at 30°C (Chromaster Ultra Rs 6310 column oven). Sugars were detected using a refractive index detector (Chromaster 5450).

Sugar contents were expressed as grams per 100 mL of fresh sample (g/100 mL). Authentic standards were obtained from Sigma–Aldrich (panose), Tokyo Chemical Industry Co. Ltd. (Tokyo, Japan) (isomaltose and isomaltotriose [IMT]), and FUJIFILM Wako Pure Chemical Corporation (Osaka, Japan) (all other sugars). All analyses were performed in triplicate.

### 2.8 Determination of α‐Amylase Activity

α‐Amylase activity was determined using a commercial enzymatic assay kit (α‐Amylase Assay Kit G7, Kikkoman Biochemifa Co. Ltd., Tokyo, Japan). Amazake samples collected at 0 h and 8 h of saccharification were centrifuged, and the supernatant was used for analysis. The reaction mixture containing substrate solution and enzyme solution was pre‐incubated at 37°C, after which the sample solution was added to initiate the reaction. After incubation at 37°C for 10 min, the reaction was terminated by adding sodium carbonate solution. The absorbance of the released chromophore was measured at 400 nm using a microplate reader. Measurements were performed in triplicate (*n* = 3), and enzyme activity was expressed as relative activity units.

### 2.9 In Vitro Antioxidant–Related Assays

These assays were conducted to characterize changes in redox–related properties during saccharification and were not intended to imply specific nutritional or health outcomes.

#### 2.9.1 2,2‐Diphenyl‐1‐Picrylhydrazyl Radical Scavenging Activity

The 2,2‐diphenyl‐1‐picrylhydrazyl (DPPH) activity was measured as previously described (Blois 1958). One gram of sample was extracted with 35 mL of 80% ethanol using a homogenizer (Bio–Gen PRO200, PRO Scientific, USA) at 7500 rpm for 15 s. Extracts were shaken at 25°C for 60 min and centrifuged (10,000 rpm, 15 min, 4°C). The supernatant (0.1 mL) was mixed with 0.9 mL of 0.1 mM DPPH solution prepared in ethanol and incubated in the dark for 30 min before absorbance measurement at 517 nm. Results were expressed as μmol Trolox equivalents per g dwb (μmol TE/g dwb).

#### 2.9.2 Ferric Reducing Antioxidant Power

The ferric reducing antioxidant power (FRAP) was measured according to the method described by Benzie and Strain (1996). The FRAP reagent (300 mM acetate buffer, 10 mM TPTZ in 40 mM HCl, and 20 mM FeCl₃·6H_2_O, 10:1:1) was prepared fresh. The sample extract (prepared for DPPH) was mixed with FRAP reagent (1:30, v/v) and incubated at 37°C for 10 min, and the absorbance at 593 nm was recorded. Results were expressed as μmol TE/g dwb.

#### 2.9.3 Total Phenolic Content

The total phenolic content (TPC) was determined using the Folin–Ciocalteu method (Folin and Ciocalteu 1927). The extracts were prepared using 80% methanol, as described above. The diluted extract (0.5 mL) was mixed with 0.1 mL of Folin–Ciocalteu reagent diluted twofold with distilled water and incubated for 3 min, followed by the addition of 0.2 mL of 10% sodium carbonate solution. After incubation for 60 min in the dark at room temperature, the absorbance was measured at 765 nm. The results were expressed as mg tannic acid equivalents per g dwb (mg TAE/g dwb).

### 2.10 Statistical Analysis

All data are presented as mean ± standard deviation unless otherwise stated. Statistical analyses were performed using SPSS Statistics v29.0.2 (IBM Corp., Armonk, NY, USA). For comparisons involving multiple processing stages (e.g., L–DOPA content), one–way analysis of variance (ANOVA) followed by Tukey's multiple comparison test was applied. For time–course data during saccharification (Brix, pH, and reducing sugars), two–way ANOVA (sample × time) followed by Tukey's test was used. Differences were considered statistically significant at *p* < 0.05. The same statistical approach was applied to antioxidant–related indices measured over time.

All experiments were performed using three independent preparation batches unless otherwise stated.

## 3 Results and Discussion

### 3.1 Proximate Composition and Micronutrient Profiles

The proximate composition, mineral content, and vitamin levels of RA and Mucuna bean amazake (MBA) were compared to evaluate differences arising from the substrate materials (Table 1). No significant differences were observed in energy or moisture contents, confirming that both samples were prepared under comparable concentration conditions. In contrast, marked differences were observed in their nutritional profiles. The protein content of MBA was approximately three times higher than that of RA, consistent with the high protein content of *M. pruriens* beans reported previously (Baby et al. 2022). Dietary fiber and crude fat contents were also significantly higher in MBA. Conversely, MBA exhibited lower carbohydrate and total sugar contents than RA.

**TABLE 1:** Proximate composition, mineral, and vitamin contents of rice amazake (RA) and Mucuna bean amazake (MBA).

	RA	MBA
*Proximate composition*		
Energy (kcal/100 g)	131 ± 1ᵃ	127 ± 2ᵃ
Water content (g/100 g)	67.2 ± 0.4ᵃ	68.0 ± 0.3ᵃ
Protein (g/100 g)	2.1 ± 0.1ᵇ	5.8 ± 0.2ᵃ
Carbohydrate (g/100 g)	30.3 ± 0.5ᵃ	25.2 ± 0.6ᵇ
Sugar (g/100 g)	29.4 ± 0.5ᵃ	23.1 ± 0.4ᵇ
Dietary fiber (g/100 g)	0.9 ± 0.1ᵇ	2.1 ± 0.1ᵃ
Crude fat (g/100 g)	0.3 ± 0.0ᵇ	0.8 ± 0.0ᵃ
Ash (g/100 g)	0.1 ± 0.0ᵇ	0.2 ± 0.0ᵃ
Salt equivalent (g/100 g)	0.003 ± 0.000ᵇ	0.010 ± 0.001ᵃ
*Minerals (mg/100 g)*		
Sodium (Na)	1.0 ± 0.0ᵇ	2.0 ± 0.1ᵃ
Potassium (K)	16 ± 1ᵇ	37 ± 2ᵃ
Calcium (Ca)	2.0 ± 0.1ᵇ	12 ± 1ᵃ
Magnesium (Mg)	4.0 ± 0.1ᵇ	11 ± 0.3ᵃ
Phosphorus (P)	24 ± 1ᵇ	46 ± 1ᵃ
Iron (Fe)	nd (< 0.01)	0.7 ± 0.0
Zinc (Zn)	0.4 ± 0.0ᵇ	0.9 ± 0.0ᵃ
Copper (Cu)	0.09 ± 0.00ᵇ	0.29 ± 0.01ᵃ
Manganese (Mn)	0.21 ± 0.01ᵇ	0.35 ± 0.01ᵃ
*Vitamins (mg/100 g)*		
Thiamine (B1)	0.01 ± 0.00ᵇ	0.05 ± 0.00ᵃ
Riboflavin (B2)	0.04 ± 0.00ᵇ	0.05 ± 0.00ᵃ
Pyridoxine (B6)	0.04 ± 0.00ᵃ	0.04 ± 0.00ᵃ
Niacin	0.4 ± 0.0ᵃ	0.4 ± 0.0ᵃ

*Note:* Values are presented as mean ± SD (*n* = 3). Different superscript letters within a row indicate significant differences between the RA and MBA groups at *p* < 0.05. nd (LOD). Statistical comparison was not performed for parameters including “nd.”

Abbreviation: nd, not detected.

Regarding micronutrient composition, MBA contained higher levels of major minerals, including K, Ca, Mg, and P, as well as trace elements such as Zn, Cu, and Mn, than RA. Iron was not detected in RA but was present in MBA (0.7 mg/100 g). These results are consistent with previous reports describing Mucuna beans as a mineral‐rich legume (Siddhuraju et al. 2000). MBA also showed significantly higher levels of vitamins B_1_ and B_2_ than RA, whereas no significant differences were observed for vitamin B_6_ or niacin. Overall, MBA was characterized by a low‐sugar, high‐protein, high‐fiber, and high‐mineral composition. These compositional differences provide a basis for interpreting the distinct saccharification behavior (Section 3.3) and subsequent changes in sugar composition and antioxidant‐related indices discussed in later sections.

The high protein content of MBA is relevant to the interpretation of its saccharification behavior, as protein–starch interactions in legume matrices are known to restrict enzymatic accessibility and thereby limit starch hydrolysis (Zhou et al. 2004). Similarly, the elevated dietary fiber content of MBA may further hinder amylase activity by physically entrapping starch granules within the cell wall matrix (Betancur‐Ancona et al. 2002). These structural features of the Mucuna bean matrix are likely to contribute to the lower saccharification efficiency observed in MBA compared with RA (Section 3.3). However, starch content and structural characteristics, including amylose/amylopectin composition, were not analyzed in the present study. Therefore, the proposed contribution of these matrix‐related factors remains inferential. In addition, the higher phenolic content expected from Mucuna beans relative to rice provides a compositional basis for the elevated antioxidant–related indices observed in MBA during saccharification (Section 3.4).

### 3.2 Reduction of L–DOPA During Preprocessing and Saccharification

**FIGURE 1:**
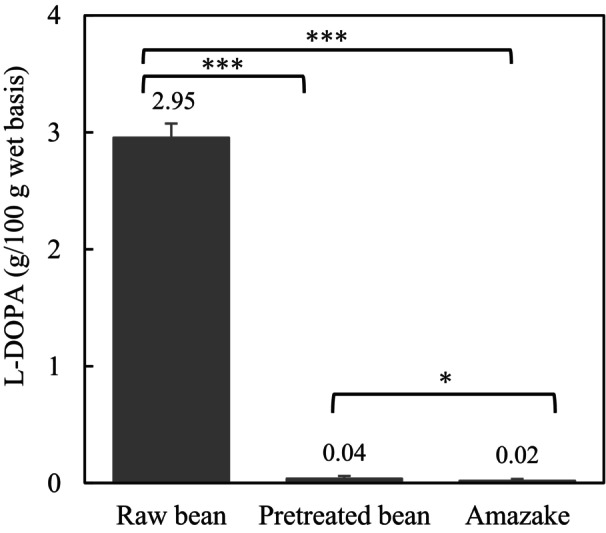
L–DOPA content of *Mucuna pruriens* beans at different processing stages. Values are presented for raw beans, prepared beans (after prolonged hot–soaking at 90°C, 16 h, dehulling, and homogenization), and amazake after 8 h of saccharification (wet basis). Error bars represent standard deviation (*n* = 6). Statistical significance was evaluated by one–way ANOVA followed by Tukey's test (*p < 0.05, ***p < 0.0001).

Changes in L–DOPA content during the production of MBA were evaluated at three processing stages: raw beans, pretreated beans (after prolonged hot–soaking at 90°C for 16 h, dehulling, and homogenization), and amazake after 8 h of saccharification (Figure 1). All values were expressed on a wet weight basis to enable direct comparison of the absolute amounts retained throughout processing.

The L–DOPA content of raw Mucuna beans was approximately 3 g/100 g, which is consistent with previously reported levels in *M. pruriens* (generally 3%–6%) (Bell and Janzen 1971; Daxenbichler et al. 1971). After prolonged hot–soaking (90°C, 16 h), dehulling, followed by homogenization, the L–DOPA level in the pretreated beans decreased to 0.04 g/100 g, corresponding to approximately 1/75 of the initial amount. L–DOPA is a highly water–soluble catechol–type amino acid derivative (Teixeira et al. 2003) and is known to leach readily into soaking or cooking water during thermal processing. In addition, homogenization disrupts cell wall structures, which may promote further release of intracellular L–DOPA into the surrounding medium.

After 8 h of saccharification, the L–DOPA concentration in the amazake was further reduced compared with that in the pretreated beans. This decrease can be partly explained by dilution resulting from the addition of rice koji and water during amazake preparation. In addition, during saccharification at approximately 55°C, L–DOPA may undergo nonenzymatic oxidation in the presence of oxygen, forming o–quinones that subsequently polymerize into melanin–like pigments. Such oxidative pathways have been reported in catechol–containing plant matrices and are likely to contribute to the additional reduction observed during saccharification.

Overall, these results demonstrate that most of the L‐DOPA originally present in Mucuna beans is effectively removed during the preprocessing steps of prolonged hot‐soaking at 90°C for 16 h, dehulling, and homogenization, and that saccharification further reduces its residual concentration in the final product. Although L‐DOPA is known as a bioactive compound in Mucuna beans, official regulatory threshold values for dietary L‐DOPA intake from foods have not been clearly established by major regulatory authorities such as the European Food Safety Authority or the U.S. Food and Drug Administration. In addition to L‐DOPA, Mucuna beans contain several other antinutritional compounds. Previous studies have shown that soaking, dehulling, and thermal processing can reduce antinutritional factors such as phytates, tannins, and protease inhibitors in legumes; therefore, similar effects may also occur during the preprocessing applied in this study, although these compounds were not evaluated. It should also be noted that naturally occurring L‐DOPA is not unique to Mucuna and has been reported in other edible legumes, including *Vicia faba* , which is recognized as a dietary source of L‐DOPA (Dhull et al. 2022). Nevertheless, further studies evaluating actual dietary exposure under practical consumption conditions would be valuable for a more comprehensive assessment of Mucuna‐based food products.

### 3.3 Saccharification Behavior of Amazake Samples

#### 3.3.1 Changes in Brix, pH, and Reducing Sugars

**FIGURE 2:**
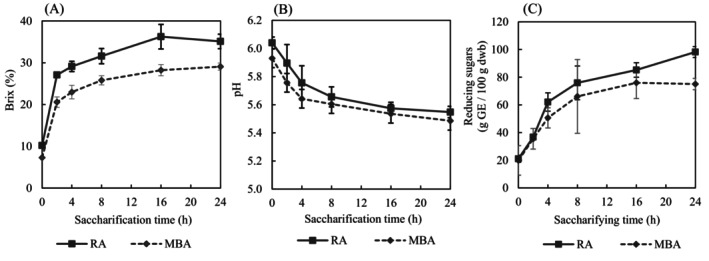
Time–course changes in saccharification indices during amazake production from rice and *Mucuna pruriens* beans. (A) Soluble solids content (Brix), (B) pH, and (C) Reducing sugar concentration (expressed as glucose equivalents by the Somogyi–Nelson method on a dry–weight basis) were monitored during saccharification at 55°C for 24 h. Error bars represent standard deviation (*n* = 6).

To clarify the saccharification characteristics of MBA, time–course changes in soluble solids (Brix), pH, and reducing sugar content, expressed as glucose equivalents using the Somogyi–Nelson method, were evaluated and compared with those of RA (Figure 2).

Both RA and MBA exhibited rapid increases in Brix values during the initial 2 h of saccharification. Thereafter, the Brix value of RA continued to increase, reaching approximately 36 °Bx at 16 h before plateauing. In contrast, MBA showed a more gradual increase, reaching approximately 29 °Bx at 24 h and remaining consistently lower than RA throughout the saccharification period. These results indicate that the accumulation of soluble sugars proceeded less extensively in MBA than in RA. Additional measurement of α‐amylase activity showed no significant difference between RA and MBA before saccharification (0 h). However, after 8 h of saccharification, α‐amylase activity decreased in RA but increased significantly in MBA (Figure S1). Therefore, the lower sugar production in MBA is more likely related to substrate characteristics than to differences in α‐amylase activity.

The pH profiles during saccharification were similar for both samples. In RA, pH decreased from approximately 6.1 at 0 h to 5.5 at 24 h, while MBA showed a comparable decrease from 6.0 to 5.45. These pH values remained within the optimal range for koji–derived amylolytic enzymes (pH 4.5–6.5) (Porfirif et al. 2016), indicating that differences in saccharification behavior between RA and MBA were not attributable to pH conditions. Changes in reducing sugar content closely mirrored the Brix profiles. In RA, reducing sugars increased continuously from approximately 20 g/100 g dwb at 0 h to nearly 100 g/100 g at 24 h, reflecting sustained starch hydrolysis and sugar production. In MBA, reducing sugars increased from approximately 17 g/100 g at 0 h to 75–80 g/100 g at 16 h, after which the levels remained nearly constant. This earlier plateau in MBA likely reflects substrate–related constraints, including lower starch availability and limited enzymatic accessibility of legume starch, which is known to exhibit higher structural resistance to hydrolysis than rice starch and is likely associated with differences in starch structure and matrix composition (Betancur‐Ancona et al. 2002; Zhou et al. 2004).

Despite these differences, both Brix and reducing sugar data demonstrate that saccharification proceeded effectively in MBA. The substantial accumulation of reducing sugars indicates that koji–derived amylases retained activity toward Mucuna–derived substrates, although the extent of sugar formation was constrained relative to that in rice–based amazake. From a food application perspective, the lower sugar formation observed in MBA may influence the perceived sweetness compared with conventional RA. However, sweetness perception depends on total sugar concentration and on sugar composition and interactions with other matrix components. Further studies including sensory evaluation will be required to clarify consumer acceptance of Mucuna‐based amazake.

#### 3.3.2 Distribution of Low–Degree–Of–Polymerization Sugars

**FIGURE 3:**
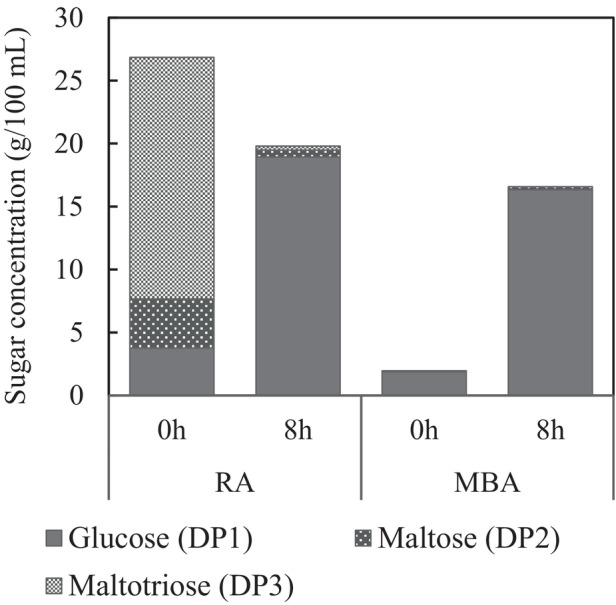
Distribution of low–degree–of–polymerization sugars (DP1–3), including glucose (DP1), maltose (DP2), and maltotriose (DP3), in rice amazake (RA) and Mucuna bean amazake (MBA) at 0 and 8 h of saccharification. Only sugars with DP ≤ 3 were quantified; higher–DP oligosaccharides were not included.

To characterize changes in sugar composition during enzymatic saccharification in greater detail, the distribution of low–degree–of–polymerization sugars (DP1–3) was analyzed before saccharification (0 h) and after saccharification (8 h) (Figure 3). The sugars examined in this analysis were glucose (DP1), maltose (DP2), and maltotriose (DP3). The concentrations of individual sugars are presented in Table S1.

In RA, maltotriose was already detected as the predominant sugar at 0 h, while glucose and maltose were also present in appreciable amounts. As saccharification progressed, the contents of maltotriose and maltose markedly decreased by 8 h, accompanied by a pronounced increase in glucose, resulting in a glucose–dominant distribution. These results indicate that rice starch was efficiently hydrolyzed by koji–derived amylases, leading to the accumulation of low–DP sugars, particularly glucose, as saccharification proceeded, which is consistent with previous reports on sugar profiles during RA production (Oguro et al. 2017; Yamashita 2021). In contrast, MBA showed a lower total content of DP1–3 sugars at 0 h than RA, with particularly low levels of maltotriose and maltose. This finding suggests that, at the early stage of saccharification, a substantial proportion of higher–DP oligosaccharides (DP ≥ 4) may be present in MBA.

Overall, although the initial sugar distribution in MBA differed from that in RA, saccharification resulted in a shift toward a low–DP sugar profile dominated by glucose in both samples. In the present study, quantitative analysis focused on sugars with DP ≤ 3. Chromatographic observations also suggested the presence of higher–DP oligosaccharides (DP ≥ 4) in the saccharified samples (Figure S2), indicating that part of the sugar fraction may consist of such oligosaccharides. Higher–DP oligosaccharides may contribute to the physicochemical and sensory properties of amazake, including viscosity and mouthfeel, and may also influence overall sweetness perception. However, their quantitative contribution remains to be clarified in future studies.

#### 3.3.3 Formation of α–1,6–Linked Oligosaccharides During Saccharification

**FIGURE 4:**
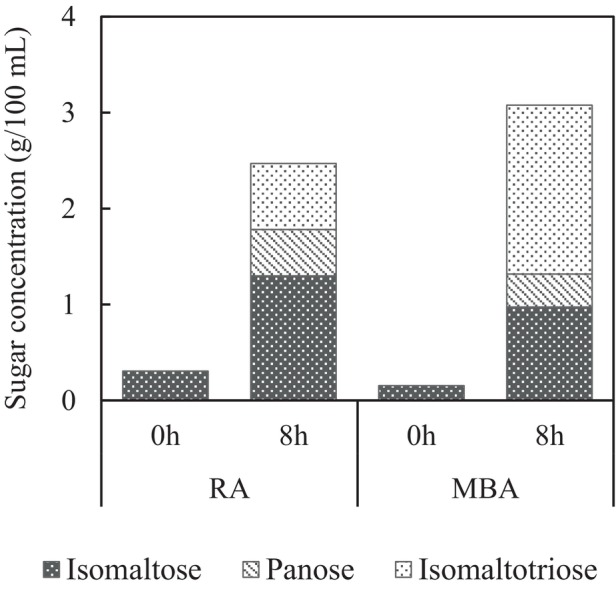
Formation of α–1,6–linked oligosaccharides during saccharification in Rice amazake (RA) and Mucuna bean amazake (MBA) at 0 and 8 h. Isomaltose, panose, and isomaltotriose (IMT) were quantified. Stacked bars represent the sum of individually quantified α–1,6–linked oligosaccharides.

To further examine qualitative changes in sugar structures during saccharification, the formation of α–1,6–linked oligosaccharides—namely isomaltose, panose, and IMT—was investigated in RA and MBA (Figure 4).

In both RA and MBA, α–1,6–linked oligosaccharides were scarcely detected prior to saccharification (0 h). After 8 h of saccharification, however, these oligosaccharides were clearly formed in both samples, with IMT and panose detected as the major components. The appearance of these sugars indicates that changes in glycosidic linkage patterns occurred during saccharification and that the observed sugar profiles cannot be explained solely by simple hydrolytic cleavage of α–1,4–linked glucans. In MBA, the total amount and composition of α–1,6–linked oligosaccharides were comparable to, and in part higher than, those observed in RA. This finding suggests that, despite the lower overall saccharification efficiency of MBA described in Section 3.3.1, reactions leading to structural rearrangement of sugars proceeded during saccharification of Mucuna–derived substrates.

The formation of isomaltose, panose, and IMT suggests the occurrence of transglycosylation reactions during saccharification. These α–1,6–linked oligosaccharides are known to be produced through transglycosylation activity of enzymes derived from *A. oryzae* in rice koji, particularly α–glucosidase and related glycosidases (Ito et al. 1997). Because the amount of rice koji used for saccharification was identical in both RA and MBA, the enzymatic system can be considered comparable between the two systems. Therefore, the observed differences in the formation of α–1,6–linked oligosaccharides are likely attributable, at least in part, to differences in substrate composition and enzymatic accessibility between rice starch and Mucuna–derived carbohydrates. Such differences in substrate structure may influence the availability of intermediate saccharides that participate in transglycosylation reactions.

It should be noted that the present study did not investigate the specific enzymatic pathways or reaction mechanisms responsible for the formation of these α–1,6–linked oligosaccharides. Therefore, the present results are reported as observed changes in sugar composition during saccharification, without further attribution to individual enzyme activities.

### 3.4 Changes in Antioxidant–Related Indices During Saccharification

**FIGURE 5:**
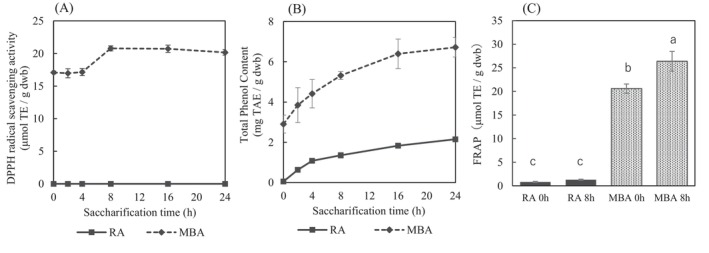
Antioxidant–related indices of rice amazake (RA) and *Mucuna* bean amazake (MBA) during saccharification. (A) DPPH radical scavenging activity expressed as μmol Trolox equivalents per g dry–weight basis (μmol TE/g dwb). (B) Total phenolic content (TPC) expressed as mg tannic acid equivalents per g dwb (mg TAE/g dwb). (C) Ferric reducing antioxidant power (FRAP) expressed as μmol TE/g dwb at 0 h and 8 h of saccharification. Solid lines and solid bars represent RA, whereas dashed lines and hatched bars represent MBA. Different letters above bars in panel (C) indicate significant differences among groups (Tukey's test, *p* < 0.05). Data are presented as mean ± SD (*n* = 3).

Changes in antioxidant–related indices of MBA and RA during saccharification were evaluated using DPPH radical scavenging activity, TPC, and FRAP (Figure 5).

DPPH radical scavenging activity (Figure 5A) was consistently higher in MBA than in RA throughout the saccharification period. MBA maintained values of approximately 4–6 μmol TE/g dwb from 8 to 24 h, whereas RA showed values close to zero at all time points. These results indicate that, despite the substantial reduction of water‐soluble phenolics such as L–DOPA during preprocessing (Section 3.2), MBA retained antioxidant‐related constituents that were not present in rice‐based amazake. A similar trend was observed for TPC (Figure 5B). MBA exhibited consistently higher TPC values than RA, with TPC increasing during saccharification and reaching approximately 7–8 mg TAE/g dwb at 24 h. In contrast, TPC in RA remained at low levels (approximately 1–2 mg TAE/g dwb) throughout the process. The gradual increase in TPC in MBA suggests that phenolic compounds associated with the bean matrix were progressively released or solubilized during enzymatic processing. This pattern is consistent with the enzymatic liberation of bound phenolics from cell wall components during hydrolysis, a mechanism reported for legume‐based substrates subjected to enzymatic treatment (Vilas‐Franquesa et al. 2023). FRAP values (Figure 5C) also differed markedly between the two samples. In MBA, FRAP increased substantially from 0 to 8 h of saccharification, reaching approximately 15–20 μmol TE/g dwb, whereas RA exhibited minimal FRAP values (< 1 μmol TE/g dwb) at both time points. Because FRAP reflects the overall reducing capacity of sample constituents, the increase observed in MBA is likely attributable to multiple reducing components generated or released during saccharification, rather than to residual L–DOPA alone. In addition to phenolic compounds, peptides and free amino acids generated through proteolytic activity during saccharification may contribute to the observed antioxidant‐related capacity, as protein‐derived fragments are known to exhibit reducing and radical‐scavenging properties (Elias et al. 2008; Zou et al. 2016). Furthermore, non‐enzymatic browning reactions at the saccharification temperature (55°C) may generate Maillard reaction products with antioxidant properties (Bolchini et al. 2025), potentially contributing to the elevated FRAP and TPC values observed in MBA.

Collectively, these results demonstrate that antioxidant–related indices in MBA remained consistently higher than those in RA during saccharification. The observed increases in TPC and FRAP likely reflect the combined release of bound phenolics, generation of antioxidant peptides, and formation of Maillard reaction products during enzymatic processing, rather than a single contributing factor. These differences are interpreted as reflecting chemical changes associated with enzymatic processing, such as the release or transformation of reducing constituents, and are not intended to indicate specific nutritional or health effects.

## 4 Conclusion

This study evaluated the fundamental characteristics of amazake produced from *M. pruriens* in comparison with conventional RA. The intrinsic L–DOPA content of Mucuna beans was markedly reduced during preprocessing, decreasing from approximately 3 g/100 g (wet basis) in raw beans to below 0.04 g/100 g, with a further reduction observed during saccharification. Although MBA exhibited lower soluble solids and reduced sugar formation than RA, time–course analyses demonstrated that saccharification proceeded steadily, indicating that Mucuna–derived starch can be effectively hydrolyzed by koji–derived enzymes despite substrate–related constraints. MBA also showed characteristic compositional changes during processing, including consistently higher antioxidant–related indices than RA. These changes are interpreted as reflecting chemical transformations occurring during enzymatic saccharification, rather than direct indicators of specific nutritional or health effects. Overall, the present findings demonstrate the feasibility of *M. pruriens* as a plant–based substrate for amazake production. The present study has several limitations that should be acknowledged. Quantitative sugar analysis was restricted to compounds with a degree of polymerization (DP) of 3 or less; higher–DP oligosaccharides detected chromatographically (Figure S2) were not quantified, and their contribution to the physicochemical and sensory properties of MBA remains to be determined. Individual phenolic compounds were not identified, and a comprehensive characterization of phenolic profiles would be required to further elucidate the origins of the elevated antioxidant–related indices observed in MBA. Amino acid and organic acid profiles were also not determined in the present study, although these aspects warrant further investigation. Furthermore, sensory evaluation was not included in the present study, and broader assessment of consumer acceptability of MBA in terms of sweetness, flavor, and texture would be valuable. Despite these limitations, the present findings provide a foundation for more comprehensive investigations of *M. pruriens* as a substrate for amazake production.

## Author Contributions

**Takako Koriyama:** conceptualization, methodology, supervision, funding acquisition, writing – review and editing, data curation, writing – original draft, visualization, project administration, formal analysis. **You Yazaki:** investigation, formal analysis, data curation.

## Funding

This work was supported by the JSPS KAKENHI (Grant Number 23K12691).

## Supporting information


**Figure S1:** Representative HPLC chromatograms of amazake samples showing sugar fractions including low‐DP sugars and higher‐DP oligosaccharides (DP ≥ 4). (A) Maltotetraose standard solution; (B) Rice amazake before saccharification (RA, 0 h); (C) Rice amazake after saccharification (RA, 8 h); (D) Mucuna bean amazake before saccharification (MBA, 0 h); (E) Mucuna bean amazake after saccharification (MBA, 8 h).
**Figure S2:** α‐Amylase activity in rice amazake (RA) and Mucuna bean amazake (MBA) before (0 h) and after (8 h) saccharification. Values are presented as mean ± SD (*n* = 3). Different letters indicate significant differences among groups (Tukey's test, *p* < 0.05).

**Table S1:** Concentrations of low–degree–of–polymerization sugars (DP1–3) and α–1,6–linked oligosaccharides in rice amazake (RA) and Mucuna bean amazake (MBA) before (0 h) and after (8 h) saccharification.

## Data Availability

The data that support the findings of this study are available on request from the corresponding author. The data are not publicly available due to privacy or ethical restrictions.
