# Supplementary material for: Enzymatic Saccharification Behavior and Compositional Characteristics of Mucuna pruriens‐Based Amazake: A Comparison With Conventional Rice Amazake
Source: Food Sci Nutr. 2026 Jul 17;14(7):e72132. doi: 10.1002/fsn3.72132 (PMC13378118; doi:10.1002/fsn3.72132)
Supplement: Supplementary file 1 — Figure S1: Representative HPLC chromatograms of amazake samples showing sugar fractions including low‐DP sugars and higher‐DP oligosaccharides (DP ≥ 4). (A) Maltotetraose standard solution; (B) Rice amazake before saccharification (RA, 0 h); (C) Rice amazake after saccharification (RA, 8 h); (D) Mucuna bean amazake before saccharification (MBA, 0 h); (E) Mucuna bean amazake after saccharification (MBA, 8 h). Figure S2: α‐Amylase activity in rice amazake (RA) and Mucuna bean amazake (MBA) before (0 h) and after (8 h) saccharification. Values are presented as mean ± SD (n = 3). Different letters indicate significant differences among groups (Tukey's test, p < 0.05). [file FSN3-14-e72132-s002.pptx]

## Slide 1
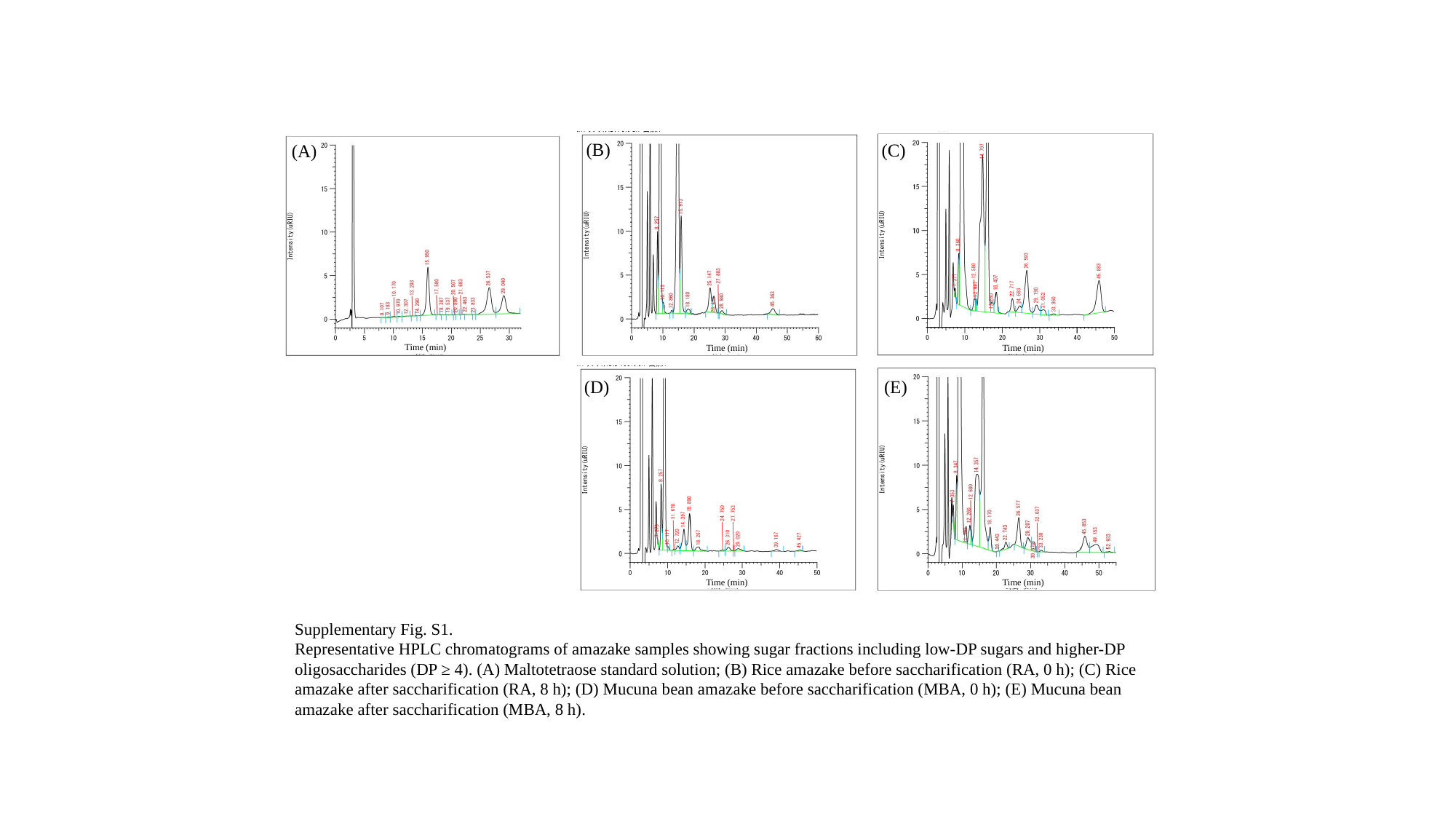

(B)
(C)
(A)
(D)
(E)
Time (min)
Time (min)
Time (min)
Time (min)
Time (min)
Supplementary Fig. S1.
Representative HPLC chromatograms of amazake samples showing sugar fractions including low-DP sugars and higher-DP oligosaccharides (DP ≥ 4). (A) Maltotetraose standard solution; (B) Rice amazake before saccharification (RA, 0 h); (C) Rice amazake after saccharification (RA, 8 h); (D) Mucuna bean amazake before saccharification (MBA, 0 h); (E) Mucuna bean amazake after saccharification (MBA, 8 h).

## Slide 2
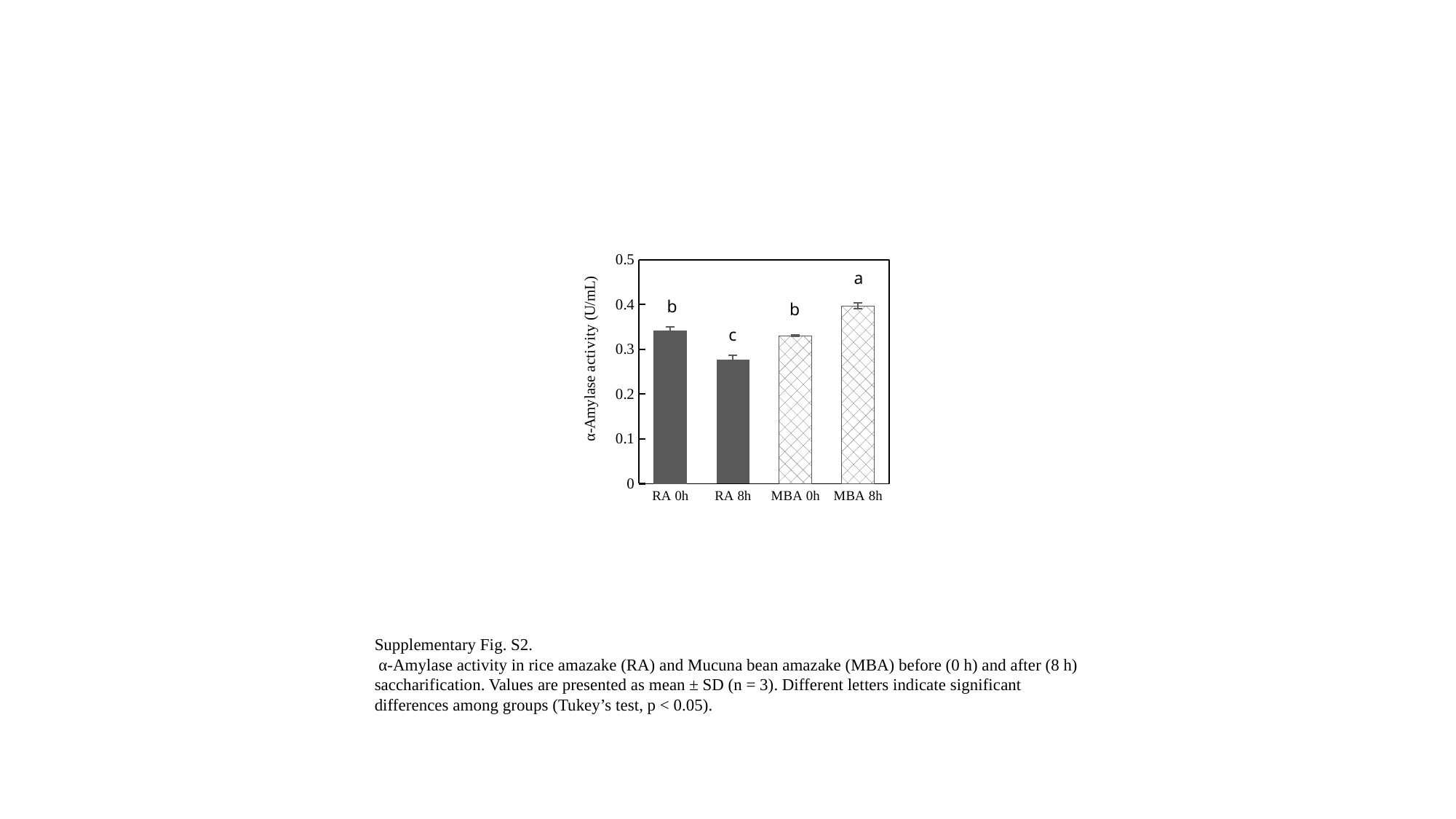

### Chart
| Category | |
|---|---|
| RA 0h | 0.34135 |
| RA 8h | 0.27709999999999996 |
| MBA 0h | 0.3302 |
| MBA 8h | 0.397 |a
b
b
c
Supplementary Fig. S2.
 α-Amylase activity in rice amazake (RA) and Mucuna bean amazake (MBA) before (0 h) and after (8 h) saccharification. Values are presented as mean ± SD (n = 3). Different letters indicate significant differences among groups (Tukey’s test, p < 0.05).
