# Supplementary material for: Enzymatic Saccharification Behavior and Compositional Characteristics of Mucuna pruriens‐Based Amazake: A Comparison With Conventional Rice Amazake
Source: Food Sci Nutr. 2026 Jul 17;14(7):e72132. doi: 10.1002/fsn3.72132 (PMC13378118; doi:10.1002/fsn3.72132)
Supplement: Supplementary file 2 — Table S1: Concentrations of low–degree–of–polymerization sugars (DP1–3) and α–1,6–linked oligosaccharides in rice amazake (RA) and Mucuna bean amazake (MBA) before (0 h) and after (8 h) saccharification. [file FSN3-14-e72132-s001.docx]

**Supplementary Table S1**

Concentrations of low–degree–of–polymerization sugars (DP1–3) and α–1,6–linked oligosaccharides in rice amazake (RA) and Mucuna bean amazake (MBA) before (0 h) and after (8 h) saccharification.

|  | RA | | MBA | |
| --- | --- | --- | --- | --- |
|  | 0h | 8h | 0h | 8h |
| Glucose (DP1) | 3.77 ± 0.2 | 18.9 ± 0.0 | 1.87 ± 0.1 | 16.3 ± 0.1 |
| Maltose (DP2) | 3.89 ± 0.1 | 0.54 ± 0.0 | 0.08 ± 0.1 | 0.25 ± 0.2 |
| Maltotriose (DP3) | 19.2 ± 5.3 | 0.33 ± 0.0 | 0.00 ± 0.0 | 0.00 ± 0.0 |
| Isomaltose | 0.31 ± 0.0 | 1.29 ± 0.1 | 0.15 ± 0.0 | 0.97 ± 0.1 |
| Panose | 0.00 ± 0.0 | 0.48 ± 0.3 | 0.00 ± 0.0 | 0.34 ± 0.2 |
| Isomaltotriose (IMT) | 0.00 ± 0.0 | 0.70 ± 0.1 | 0.00 ± 0.0 | 1.76 ± 0.2 |

Values are expressed as g/100 mL of fresh sample and are presented as means ± standard deviation (n = 3).

Values of 0.00 indicate concentrations below the detection limit of the HPLC method.
